# Large language models and their applications in bioinformatics

**DOI:** 10.1016/j.csbj.2024.09.031

**Published:** 2024-10-05

**Authors:** Oluwafemi A. Sarumi, Dominik Heider

**Affiliations:** a University of Münster, Institute of Medical Informatics, Albert-Schweitzer-Campus, Münster, 48149, Germany; b Institute of Computer Science, Heinrich-Heine-University Duesseldorf, Graf-Adolf-Str. 63, Duesseldorf, 40215, Germany

**Keywords:** Bioinformatics, Large language models, Natural language processing, Omics data

## Abstract

Recent advancements in Natural Language Processing (NLP) have been significantly driven by the development of Large Language Models (LLMs), representing a substantial leap in language-based technology capabilities. These models, built on sophisticated deep learning architectures, typically transformers, are characterized by billions of parameters and extensive training data, enabling them to achieve high accuracy across various tasks. The transformer architecture of LLMs allows them to effectively handle context and sequential information, which is crucial for understanding and generating human language. Beyond traditional NLP applications, LLMs have shown significant promise in bioinformatics, transforming the field by addressing challenges associated with large and complex biological datasets. In genomics, proteomics, and personalized medicine, LLMs facilitate identifying patterns, predicting protein structures, or understanding genetic variations. This capability is crucial, e.g., for advancing drug discovery, where accurate prediction of molecular interactions is essential. This review discusses the current trends in LLMs research and their potential to revolutionize the field of bioinformatics and accelerate novel discoveries in the life sciences.

## 1 Introduction

Large Language Models (LLMs) [1] [2] [3] represent a pivotal advancement in natural language processing (NLP), characterized by their ability to handle complex linguistic tasks with remarkable accuracy and versatility. These models, such as the Generative Pre-trained Transformer (GPT) [4] and Bidirectional Encoder Representations from Transformers (BERT) models [5], are designed to understand and generate human-like text by leveraging vast amounts of pre-existing textual data. The key strength of LLMs lies in their architecture, which typically includes transformer-based [6][7] frameworks that excel at capturing intricate language patterns and dependencies across long distances within sentences. This architecture allows LLMs to handle various NLP tasks simultaneously, improving efficiency and broadening their application scope.

Through pre-training on massive corpora [8], LLMs acquire a deep contextual understanding of language, enabling them to perform various tasks, from text generation and sentiment analysis to machine translation and summarization. Additionally, LLMs are usually fine-tuned [9] on specific tasks to adapt their general language knowledge to more specialized domains, ensuring robust performance across diverse NLP applications. This fine-tuning process allows for customizing models to cater to specific industry needs, such as legal document analysis, customer service automation, and more, highlighting the transformative impact of large-scale data-driven models in advancing artificial intelligence capabilities in understanding and generating natural language.

In addition to traditional NLP tasks, LLMs have shown significant promise in bioinformatics. Bioinformatics, which involves the application of computational tools to process biological data, has greatly benefited from the capabilities of LLMs. These models are proving invaluable due to their ability to handle large datasets and process complex information, which are common challenges in this domain. By leveraging the use of transformers, LLMs can analyze vast amounts of biological data, providing researchers with powerful tools to uncover insights that were previously out of reach.

LLMs have demonstrated significant potential in advancing drug discovery [10], gene expression analysis [11], and biological pathway analysis [12]. Similarly, LLMs have played a crucial role in predicting protein structures [13] [14], leveraging their NLP abilities to forecast intricate three-dimensional arrangements of proteins. Such predictions hold immense promise for drug development and unraveling protein functions across diverse biological pathways.

Additionally, LLMs prove invaluable in genomics analysis [15] [16] by deciphering DNA and RNA sequences, pinpointing genetic alterations, and anticipating their implications on protein behavior. This accelerated approach fuels research on genetic disorders and fosters advancements in personalized healthcare. LLMs also excel in extracting pertinent information from scientific texts [17], identifying promising drug targets [18], and foreseeing drug-biological target interactions [19]. Furthermore, LLMs have significantly contributed to disease classification [20] by analyzing genetic and proteomic data to identify biomarkers and classify disease states more accurately. In precision medicine [21], LLMs facilitate the tailoring of medical treatments to individual genetic profiles, enhancing the effectiveness of therapies and reducing adverse effects.

In recent years, several LLM tools for bioinformatics applications have emerged as shown in Table 1. This rise in LLM tools is driven by the need for specialized models tailored to specific bioinformatics tasks, unlike general-purpose LLMs. The ability to fine-tune these models for distinct bioinformatics tasks has significantly contributed to their rapid adoption and development. These task-specific LLMs, trained on relevant biological datasets, enable more precise and efficient problem-solving, advancing genomics, proteomics, drug discovery, and precision medicine. As LLMs continue to drive cutting-edge research in bioinformatics, there is a growing need to summarize the current applications of these models in bioinformatics, highlight their transformative potential, and identify areas for further research and improvement. This review provides a concise, yet comprehensive overview of the present state of LLMs in bioinformatics applications, serving not only to track progress but also as a springboard for future developments in this fast-evolving field.

**Table 1:** LLMs Tools for Bioinformatics Applications.

Bioinformatics Task	Model Name	Year	Base Models	Research Direction
Protein Structure Prediction	ESM-1b	2019	ESM	Biological structures analysis.
	EpiBERTope	2021	BERT	Identification of linear and structural epitopes.
	ProtTrans	2021	BERT, Albert, Electra, T5	Prediction of protein structure and function.
	ESM-1V	2021	ESM	Predicting effects of mutations on protein function.
	ProtGPT-2	2022	GPT-2	Optimization of protein design, protein function prediction.
	ESMFold	2022	ESM	Protein folding mechanisms and interactions.
	ProteinBERT	2022	BERT	Prediction of protein-protein interactions.

Biological Sequence Analysis	DNABERT	2021	BERT	Prediction of complex DNA patterns.
	GeneBERT	2021	BERT	Regulatory genome modeling.
	TCR-BERT	2021	BERT	Understanding and predicting TCR-antigen interactions.
	RNABERT	2022	BERT	RNA classification, functional annotation, and mutation effect prediction.
	MetaBERTa	2023	RoBERTa	Taxonomic classification of metagenomic data.
	HyenaDNA	2023	Hyena	Modeling of genomic data across various scales.
	DNAGPT	2023	GPT	Sequence analysis such as gene annotation, variant calling, and motif discovery.
	DNABERT-2	2024	BERT	Motif detection, and cross-species genomic comparisons.
	DNABERT-S	2024	BERT	Sequence classification and motif detection.

Drug Discovery	SMILES-BERT	2019	BERT	Predicting molecular properties from chemical structure representations.
	ChemBERTa	2020	RoBERTa	Molecular property predictions and bioactivity classifications.
	K-BERT	2022	BERT	Molecular data analysis
	TransDTI	2022	ESM, ProtBert	Estimating drug-target interactions
	MolGPT	2022	GPT	Generation of diverse and chemically valid molecules.
	PharmBERT	2023	BERT	Enhanced drug safety monitoring, and interaction

Gene Expression Analysis	GeneBERT	2021	RoBERTa	Differential gene expression using histone modification data.
	scBERT	2022	BERT	Relationships in gene expression profiles and cell types.
	iEnhancer-BERT	2022	BERT	Identification of enhancers and their functional strength
	MuLan-Methyl	2022	BERT, DistilBERT, ALBERT, XLNet, ELECTRA	Identification of methylation sites across different genomic contexts.
	DeepGeneT	2024	—	Classification of lung cancer subtypes.

Pathway Analysis	PathNER	2013	—-	Extraction of biological pathways from scientific literature.
	BioBERT	2019	BERT	Biomedical text mining tasks.
	ClinicalBERT	2019	BERT	Improved medical domain results using clinical specific contextual embeddings.
	Lomics	2024	LLama-3	Generation of pathways and gene sets for transcriptomic analysis.
	Galactia	2024	—	Identification of protein interactions, pathways, and gene regulation.

## 2 LLMs models in bioinformatics domain

Large language models like BERT and GPT-series have been tailored for bioinformatics purposes, resulting in specialized models such as DNABERT [22], ProteinBERT [23], DNAGPT [24], and ProGPT2 [25]. These adaptations exhibit promising capabilities in deciphering extensive unstructured biological datasets, empowering researchers to derive significant insights and drive informed decision-making. In DNABERT, the authors adapted the BERT model for genomic sequences by conceptualizing DNA as a language using k-mers. To handle the unique structure of DNA, DNABERT tokenizes sequences into k-mers (subsequences of length k), treating these as analogous to words in natural language processing. This allows the model to learn bidirectional representations of nucleotide sequences, considering both upstream and downstream contexts simultaneously. Pre-trained on extensive genomic datasets, DNABERT effectively captures complex DNA patterns and excels in tasks such as predicting transcription factor binding sites through fine-tuning. The model significantly outperforms traditional methods in genomic benchmarks and provides interpretable results via its attention mechanisms, highlighting crucial regions in DNA sequences.

DNABERT-2 [26] provided an enhanced version of the original DNABERT model, designed for multi-species genomic analysis. This model builds on the BERT architecture to efficiently capture and represent DNA sequences from various species, improving upon its predecessor with performance and computational efficiency optimizations. DNABERT-2 introduces optimized pretraining techniques, such as masked k-mer language modeling, where k-mers are masked and predicted, allowing the model to learn deeper representations of nucleotide sequences. The structure also includes layers fine-tuned to better handle the intricacies of genomic data, with improvements in handling sequence diversity and capturing rare variants. Additionally, DNABERT-2 employs larger, more diverse genomic data, for pretraining, which enhances its generalization to various genomic tasks, allowing it to excel in sequence classification, motif detection, and cross-species genomic comparisons.

The authors in DNABERT-S [27] proposed an advanced genomic model incorporating species-specific information into DNA sequence analysis. By integrating species-aware embeddings and undergoing enhanced pre-training on diverse genomic data, DNABERT-S excels in tasks like sequence classification and motif detection, outperforming previous models and traditional methods. Its ability to provide interpretable results via attention mechanisms makes it valuable for comparative genomics and evolutionary studies. This model marks a significant advancement in genomic research, emphasizing the importance of species-specific information for more accurate and insightful analyses.

Furthermore, in RNABERT [16], the authors presented a deep learning-based method for embedding RNA bases, enabling effective structural alignment and clustering of RNA sequences. The model uses multiple layers of self-attention mechanisms to capture long-range dependencies and contextual relationships within RNA sequences. Pretrained on large RNA datasets, RNABERT leverages bidirectional encoding to analyze RNA sequences both upstream and downstream simultaneously, making it highly effective in tasks such as RNA classification, functional annotation, and mutation effect prediction. Its architecture allows it to learn deep, context-aware representations of RNA, which improves accuracy in downstream tasks. This technique improves upon traditional alignment methods, offering enhanced accuracy and efficiency. The results demonstrate the model's ability to provide meaningful RNA embeddings, which can be applied to various RNA analysis tasks.

Also, a deep learning model designed to predict protein sequences and functions by leveraging the BERT architecture was proposed in ProteinBERT [23]. ProteinBERT is pre-trained on vast protein sequence data, capturing intricate sequence patterns and biological features. This model demonstrates versatility across a wide range of protein-related tasks, such as predicting protein-protein interactions, subcellular localization, and functional annotations. The model's ability to provide interpretable insights into protein functions and interactions underscores its potential as a powerful tool in computational biology and bioinformatics.

Generally, the BERT model key approach lies in its bidirectional architecture, which allows it to consider both the left and right contexts of a word simultaneously during training. BERT is pre-trained using two tasks–masked language modeling, where random words in a sentence are masked and predicted, and next sentence prediction, which helps the model understand sentence relationships. BERT relies only on the transformer's encoder stack, which processes the entire input sequence at once, making it highly effective for tasks such as classification. By leveraging full sequence information in both directions, BERT excels at capturing intricate patterns and context within genomic data.

Additionally, LLM models for bioinformatics applications based on the GPT series have shown promising potential. GPT which was originally designed for language generation tasks, adopts a unidirectional approach. It uses the transformer's decoder stack, which processes input sequences from left to right (autoregressively), predicting the next word based only on previous tokens. This unidirectional nature makes GPT particularly well-suited for text generation, where each word is generated sequentially. DNAGPT [24], inspired by the GPT models, utilizes extensive pre-training on large DNA datasets, enabling it to efficiently handle a variety of bioinformatics tasks, such as gene annotation, variant calling, and motif discovery. The model treats DNA sequences as a continuous language, applying transformer-based frameworks to capture complex genomic patterns. Its versatility lies in its ability to generalize across multiple bioinformatics tasks, similar to other transformer architectures. By streamlining intricate sequence analysis and improving prediction accuracy, DNAGPT represents a significant leap in genomics, with far-reaching implications for both genetic research and molecular biology.

ProGen2 [28] demonstrated that LLMs, trained on extensive datasets of protein sequences, can accurately produce functional proteins across various protein families. The study highlights the model's ability to capture complex sequence patterns and relationships, enabling the generation of novel protein sequences that exhibit desired structural and functional characteristics. The research validates the efficacy of LLMs through experimental assays, showing that the generated proteins not only maintain functionality but also diversify the existing protein repertoire.

ProtGPT2 [25] designed for generating and understanding protein sequences, consists of multiple transformer layers, utilizing self-attention mechanisms to model the dependencies between amino acids in protein sequences. ProtGPT2 tokenizes protein sequences into individual amino acids, and pretrained on large protein sequence databases, learning to predict the next amino acid in a sequence, which enables it to generate novel protein sequences. ProtGPT2 is built on the autoregressive nature of GPT-2, which allows it to generate sequences by iteratively predicting the next token based on prior context. ProtGPT2 is optimized for protein design, protein function prediction, and understanding sequence-structure relationships in proteins. This model marks a significant advancement in protein design, offering a powerful tool for researchers in bioinformatics and synthetic biology to explore new protein structures and functions.

## 3 Application of LLMs in bioinformatics

**Fig. 1:**
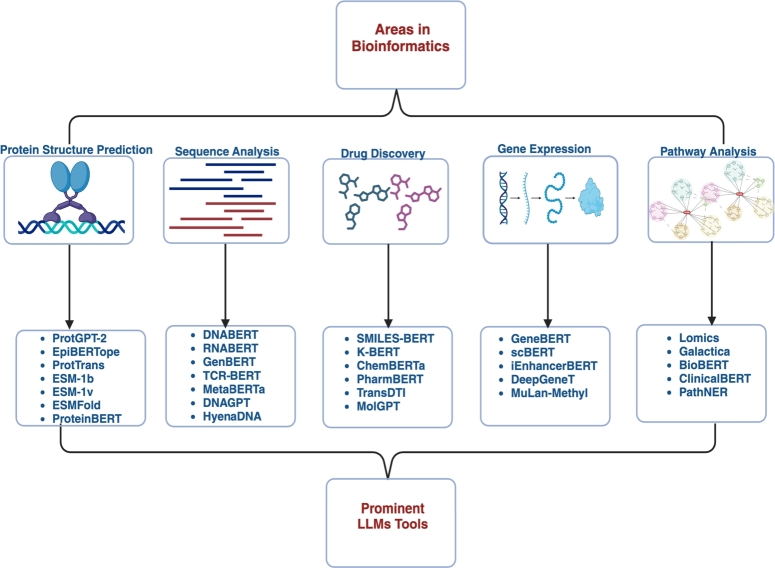
Visualization of LLMs tools developed to improve research activities in various bioinformatics application areas. The figure was created with Biorender.com.

LLMs tools have been developed and applied to various areas in bioinformatics as shown in Fig. 1. This integration of LLMs in bioinformatics is reshaping the approach to biological data analysis and accelerating discoveries in the life sciences. These models help streamline the data interpretation process, making it possible to quickly derive meaningful insights from complex datasets. As a result, applying LLMs is driving significant advancements in understanding biological processes and developing new therapeutic strategies. The ongoing development and refinement of LLMs promise to revolutionize bioinformatics further, pushing the boundaries of what can be achieved in biological research and medical practice.

### 3.1 Protein structure prediction

LLMs have become a versatile model for bioinformatics applications, especially in protein structure prediction. These advanced models facilitate the understanding of protein folding by analyzing extensive datasets of protein sequences and accurately predicting their three-dimensional structures. AlphaFold [29], primarily based on a combination of deep learning techniques, including convolutional neural networks and attention mechanisms represents a breakthrough in predicting protein structures, greatly enhancing our comprehension of protein folding and function. Similarly, ProtGPT-2 [25] showcases the versatility of LLMs by generating and refining protein structures, highlighting their utility in protein engineering. LLMs also play a crucial role in identifying potential drug targets within protein structures. By leveraging their sophisticated natural language processing capabilities, these models can sift through large volumes of biological data to pinpoint critical regions of proteins that may serve as therapeutic targets. Also, Codex [30], adapted for bioinformatics applications, exemplifies this capability by assisting in identifying binding sites and designing molecules that interact with these sites effectively.

Furthermore, EpiBERTope [31], a pre-trained BERT model designed for predicting linear and structural epitopes in proteins enhances epitope prediction by learning long-distance interactions within protein sequences, providing more accurate identification of epitopes and advancing our understanding of protein-antibody interactions. Additionally, ProtTrans [13], a deep learning model that uses self-supervised learning and high-performance computing to decode protein sequences captures complex patterns and relationships within protein sequences, significantly advancing bioinformatics and offers profound insights into protein biology and molecular research.

Additionally, ESM-1b [32] is a transformer-based protein language model designed for various applications in protein biology, including predicting protein structures and functions. As a foundational model, ESM-1b has inspired variants like ESM-1v [33] and ESM-IF1 [34], tailored for specific tasks. ESM-1v predicts the effects of mutations on protein function, analyzing how single amino acid changes influence structural stability and activity by using attention mechanisms to identify key residues. In contrast, ESM-IF1 focuses on inverse folding, deriving sequences that can adopt specific target structures, aiding in protein design and engineering. Building on these advancements, ESMFold [35] employs transformer architectures to predict protein structures from amino acid sequences. By utilizing a sequence-to-structure paradigm and pre-training on large datasets, ESMFold generates accurate 3D models, enhancing understanding of protein folding mechanisms and interactions.

### 3.2 Biological sequence analysis

LLMs have found versatile applications in biological sequence analysis, revolutionizing tasks such as DNA sequence classification, gene prediction, and RNA structure analysis. Leveraging their advanced natural language processing capabilities, these models have transformed the interpretation and analysis of biological sequences. In DNA sequence classification, models like DNABERT [22] can accurately categorize sequences, identify gene functions, and predict the effects of genetic mutations. This ability to anticipate the potential consequences of genetic alterations could assist in better predictions of disease risk, identification of pathogenic variants, and the development of targeted therapeutic strategies. Also, by understanding the contextual relationships between nucleotides, DNABERT can recognize sequence patterns that are indicative of specific biological functions, aiding researchers in exploring gene functions more comprehensively. In gene prediction, LLMs such as GeneBERT [36] have been used to predict gene locations and functions, assisting in annotating genomes and understanding gene regulation. These models analyze vast datasets to identify patterns and sequences indicative of specific gene functions, significantly enhancing our knowledge of genetic coding.

Additionally, RNA structure analysis has particularly benefited from LLMs. Models, such as RNABERT [16], have shown considerable potential in unraveling the intricate folding patterns of RNA molecules. By leveraging their capability to understand and process complex sequences, researchers can delve deeper into the complexities of RNA structures and their functional implications. This capability is crucial for elucidating fundamental biological processes such as gene regulation, protein synthesis, and various cellular mechanisms. RNABERT's ability to analyze and predict RNA structures could enable scientists to explore RNA's role in gene expression and its interactions with other biomolecules.

Also, TCR-BERT [37]is a specialized model designed to analyze T-cell receptors (TCRs) by learning their “grammar” through advanced natural language processing techniques, leverages the BERT architecture to understand and predict TCR-antigen interactions, enabling more flexible and accurate analyses of antigen binding. By training on extensive datasets of TCR sequences and their associated antigens, TCR-BERT enhances the ability to decipher complex binding patterns and interactions, facilitating improved antigen recognition and immune response studies.

In MetaBERTa [38], the authors proposed an approach to tackling the complexities involved in processing metagenomic data by implementing the RoBERTa [2] LLM variant for metagenomic applications. Given the challenges posed by the immense genetic diversity in microbiome sequencing data, the authors pre-train MetaBERTa on extensive genomic datasets to generate embeddings that facilitate the analysis of intricate metagenomic information. The study evaluates the effectiveness of these embeddings in taxonomic classification across three distinct datasets and demonstrates that the model enhances the interpretability and utility of metagenomic analyses.

Furthermore, HyenaDNA [39], which is specifically tailored to handle long-range dependencies in genomic sequences, utilizes a combination of efficient linear attention mechanisms and dynamic convolutions to process DNA sequences at single-nucleotide resolution. The model integrates a hierarchical structure that allows it to capture both short-range and long-range interactions between nucleotides, which is crucial for understanding genomic data across various scales. By employing such mechanisms, HyenaDNA can model sequences over thousands of base pairs, addressing the limitations of traditional transformers in terms of memory and computational efficiency.

### 3.3 Drug discovery

LLMs have revolutionized drug discovery processes by leveraging their sophisticated capabilities to analyze extensive repositories of biomedical literature and molecular datasets. These models have become indispensable tools for identifying promising drug targets and predicting interactions between drugs and biological targets by harnessing their natural language understanding abilities. For example, models like BERT and GPT-3 have been used to extract relevant information from vast biomedical databases, aiding in discovering new drug candidates and understanding complex disease mechanisms.

Integrating LLMs into bioinformatics has significantly advanced the field, enhancing the capacity to derive valuable insights from intricate biological datasets. These models facilitate the extraction of nuanced information from complex biological contexts, empowering researchers with a deeper understanding of the underlying mechanisms governing biological processes. The authors K-BERT [40], a method designed to extract molecular features in a manner akin to computational chemists presents an approach that leverages the BERT architecture to analyze and interpret molecular data. By incorporating domain-specific knowledge, the model achieves a fine-grained understanding of molecular structures and their properties, enabling it to derive features that are both relevant and insightful for various bioinformatics applications.

In SMILES-BERT [41], an approach for molecular property prediction using large-scale unsupervised pre-training on Simplified Molecular Input Line Entry System (SMILES) strings. The authors adapted the BERT model to understand and predict molecular properties from chemical structure representations. By leveraging a vast corpus of unlabeled SMILES strings, SMILES-BERT learns meaningful representations of molecules, which are then fine-tuned for various downstream tasks, such as predicting molecular properties and bioactivities. The authors demonstrate that SMILES-BERT significantly outperforms traditional methods and other deep learning approaches, highlighting its potential to advance computational chemistry and drug discovery through improved molecular property predictions.

Also, ChemBERTa [42], a model designed for large-scale self-supervised pretraining specifically tailored for molecular data leveraged the RoBERTa architecture to encode chemical information from SMILES strings, which represent molecular structures. ChemBERTa learns to capture the intricate features and relationships within molecular data by training on extensive unlabeled molecular datasets. The authors demonstrate that ChemBERTa, once pre-trained, can be fine-tuned for various downstream tasks such as molecular property prediction and bioactivity classification.

In PharmBERT [43], a specialized BERT model pre-trained on human prescription drug labels and fine-tuned to effectively process and interpret drug labels. Drug labels, which include prescribing information, patient guides, and packaging details, contain crucial data such as pharmacokinetics, adverse events, and drug interactions. By leveraging this domain-specific information, PharmBERT can enhance the accuracy and efficiency of tasks related to drug safety monitoring, adverse reaction detection, and drug interaction identification, making it a valuable tool for improving pharmacovigilance and regulatory compliance.

Additionally, TransDTI [44], presents a transformer-based language model for estimating drug-target interactions (DTIs) and building an effective drug recommendation workflow. The work applies transformer architecture on large-scale molecular data to learn and predict interactions between drugs and their targets with high accuracy. The authors argued that the proposed model can identify potential drug-target pairs and suggests a robust framework for drug discovery and repurposing.

Furthermore, MolGPT [45], a novel approach for molecular generation utilizing a transformer-decoder model explored the capabilities of the GPT architecture to create novel molecular structures. By training the model on a large dataset of molecular sequences, MolGPT can generate diverse and chemically valid molecules with potential applications in drug discovery and material science. The authors demonstrate that MolGPT excels in generating molecules that meet specific desired properties, outperforming traditional methods and other deep learning models. These advancements drive significant progress in healthcare and therapeutic interventions, facilitating the development of new treatments and improving patient outcomes.

### 3.4 Gene expression analysis

The ability of LLMs to integrate and analyze gene expression data extends to understanding the underlying mechanisms that drive cellular processes and disease states. LLMs can identify co-expressed gene modules and predict gene interactions based on expression patterns, providing a deeper understanding of gene regulatory networks [46] [47]. This capability is crucial for deciphering how gene expression changes influence cellular functions and contribute to disease progression. By revealing intricate details about gene regulation, such as identifying novel transcription factors or signaling pathways that modulate gene activity, LLMs help researchers pinpoint potential therapeutic targets and biomarkers.

In cancer research [48], LLMs have been used to analyze differential gene expression profiles between tumor and normal tissues to uncover aberrantly regulated genes that may serve as targets for new treatments [49]. In DeepGene Transformer [50] transformer models were utilized to classify lung cancer subtypes based on gene expression data. The work utilized the self-attention mechanism of transformers to effectively handle and interpret the complex, high-dimensional data characteristic of gene expression profiles.

Similarly, in developmental biology [51], LLMs can assist in understanding how gene expression changes during development impact cellular differentiation and tissue formation. scBERT [52] employs a large-scale pretraining approach, adapting the architecture of BERT to training on a vast array of single-cell datasets, and capturing intricate patterns and relationships in gene expression profiles and cell types. The authors demonstrate that scBERT not only improves annotation quality but also offers robustness across diverse datasets and experimental conditions.

GeneBERT [53], an application of the RoBERTa architecture for predicting differential gene expression using histone modification data. GeneBERT captures complex patterns in histone modifications that correlate with gene expression changes. This approach highlights the potential of transformer models to enhance the analysis and interpretation of high-dimensional biological data, offering deeper insights into gene regulation mechanisms and the impact of epigenetic modifications on gene expression.

Also, iEnhancer-BERT [54] introduces a transfer learning method based on DNABERT, a pre-trained DNA language model on the human genome. This model integrates a BERT layer for deep feature extraction with a convolution neural network layer for classification, employing transfer learning to fine-tune its understanding of enhancer sequences. Unlike traditional fine-tuning, iEnhancer-BERT forms its feature vector by extracting outputs from all Transformer Encoder layers, enhancing its ability to identify enhancers and their functional strength. This approach advances LLM in genomics by providing a more refined tool for understanding gene expression regulation.

Furthermore, MuLan-Methyl [55] employs multiple transformer-based large language models to improve the accuracy of DNA methylation prediction. By harnessing the power of transformer architectures, it captures the intricate patterns and complexities of DNA methylation—a crucial process in gene regulation with significant implications for diseases such as cancer. The model uses a “pretrain and fine-tune” approach, where pretraining occurs on a custom corpus of DNA fragments with self-supervised learning, followed by fine-tuning to predict methylation status. By integrating multiple transformer models, MuLan-Methyl enhances the identification of methylation sites across different genomic contexts, leading to more precise epigenetic analysis. This tool represents a key contribution in bioinformatics, offering researchers enhanced capabilities for understanding the epigenetic landscape and its role in health and disease.

### 3.5 Pathway analysis

LLMs have become a useful tool in biological pathway analysis [56] by exploiting their advanced natural language processing capabilities to integrate and interpret complex biological data. They have shown promising potential in processing and analyzing vast amounts of scientific literature and biological data, enabling researchers to map out intricate biological pathways and identify vital regulatory components. Lomics [57], adapted LLMs to enhance pathway and gene set analysis in transcriptomic studies. By adapting transformer-based LLMs for biological data, Lomics significantly improves the accuracy and depth of identifying biologically relevant pathways and gene sets. The tool integrates transcriptomic data with other omics layers, providing a comprehensive view of gene expression patterns and their regulatory mechanisms. Lomics could facilitate the understanding of complex gene interactions and the discovery of disease mechanisms and therapeutic targets.

Additionally, Galactica [58] is an LLM tool for automating the extraction of molecular interactions and pathway knowledge from scientific literature. The authors highlight Galactica's advanced capabilities in accurately identifying and extracting pathway-related information. By integrating Galactica into bioinformatics pipelines, the study showcases the model's potential to advance research in genomics and molecular biology through improved comprehension of complex biological processes.

In BioBERT [59], a pre-trained biomedical language model fine-tuned on a large corpus of biomedical literature, primarily designed for text mining tasks across various biomedical domains, offers significant advantages for biological pathway analysis by facilitating the extraction of biological entities such as genes, proteins, and diseases and their relationships from scientific literature. BioBERT's ability to process and identify complex biomedical terms could enhance the iscovery of novel connections within biological pathways, improving the predictive modeling of functional relationships between biological components. This will not only help in constructing more detailed pathway maps but also aids in the interpretation of molecular mechanisms in diseases, offering valuable insights for precision medicine and drug discovery.

Also, ClinicalBERT [60] shows enhanced performance when fine-tuned for specific note types in clinical texts. This improvement optimizes the extraction of biomedical entities, such as gene pathways and molecular interactions. Leveraging domain-specific models like ClinicalBERT enable researchers to more precisely identify molecular pathways involved in disease processes. This refined analysis deepens the understanding of underlying biological mechanisms, enhances pathway analysis, and offers valuable insights into the molecular foundation of various diseases.

A specialized text-mining tool designed to automatically identify and extract biological pathways from scientific literature was presented in PathNER [61]. PathNER leveraged NLP techniques to systematically recognize pathway-related entities, facilitating the curation of pathway databases and supporting the reconstruction of metabolic networks. By accurately identifying pathway mentions, PathNER aids researchers in navigating the extensive and complex biomedical literature, thereby enhancing the efficiency and comprehensiveness of biological pathway analysis. The authors demonstrate the effectiveness of PathNER through its application to a large corpus of biomedical texts, highlighting its potential to significantly streamline the extraction and organization of pathway information for further biological research and analysis.

## 4 Challenges and future directions

Although the potential applications of large language models in bioinformatics hold great promise, several challenges must be addressed to harness their benefits entirely. Top among these challenges are model interpretability, data bias, ethical considerations, and hallucinations of LLMs-based tools. Interpretability [62] remains a critical issue, as the inner workings of large language models can often seem opaque and difficult to comprehend. Understanding how these models arrive at their predictions is essential for ensuring the reliability and trustworthiness of their outputs, especially in sensitive domains like healthcare and biological research. Moreover, data bias [63] poses a significant obstacle, as large language models trained on biased datasets may perpetuate or even exacerbate existing biases in their predictions and recommendations. Addressing this issue requires careful curation and diversifying training data to ensure that models generate fair and unbiased outputs across diverse populations and contexts.

Ethical considerations [64] must also be properly taken into consideration, addressing issues concerning data privacy, consent, and potential misuse of AI-powered technologies in biological research. Safeguarding patient privacy and upholding ethical data collection, storage, and analysis standards are paramount to ensuring the responsible and ethical use of large language models in bioinformatics.

Also, the issue of hallucination in LLMs [65][66], where the models generate false or misleading information, must be carefully addressed in bioinformatics applications where accuracy and reliability are paramount, such as in drug discovery and gene expression analysis. Given the complexity of biological data and the potential consequences of errors, it is essential to integrate domain expertise and implement rigorous validation protocols when using LLM tools. Ensuring that outputs are critically evaluated by experts and cross-validated with trusted data sources can help mitigate the risks associated with hallucinations, thereby improving the reliability and safety of LLM-driven bioinformatics research.

Future research efforts should prioritize the development of specialized language models explicitly tailored for bioinformatics applications. By optimizing model architectures and training methodologies to suit the unique requirements of biological data analysis, researchers can enhance the accuracy, interpretability, and ethical integrity of large language models in bioinformatics. Additionally, more efforts should be exerted in fostering interdisciplinary collaborations between computer scientists, bioinformaticians, ethicists, and policymakers to develop guidelines, frameworks, and regulatory measures that promote responsible and ethical AI-driven research practices in bioinformatics.

## 5 Conclusion

The transformative potential of LLMs in bioinformatics is undeniable, encompassing various applications from natural language processing to biological sequence analysis. As evidenced by their remarkable performance in tasks such as protein structure prediction, genomics data analysis, and drug discovery, these models have demonstrated their versatility and efficacy in tackling complex biological challenges. Continued research and development efforts promise to unlock even greater capabilities in LLMs, further enhancing their impact on bioinformatics. By refining model architectures, expanding training datasets, and exploring innovative applications, researchers can harness the full potential of these powerful tools to deepen our understanding of biological systems and accelerate the pace of scientific discovery.

Moreover, integrating LLMs into bioinformatics workflows can revolutionize the way we approach biomedical research and drug development. By providing researchers with unprecedented access to vast amounts of biomedical literature, molecular data, and genomic sequences, these models empower them to uncover hidden patterns, identify novel insights, and make informed decisions with greater precision and efficiency. Ultimately, the future of bioinformatics is intricately linked with the continued advancement and adoption of LLMs. With their transformative capabilities and boundless potential, these models are poised to reshape the landscape of biological research, driving breakthroughs in understanding disease mechanisms, discovering new therapeutic targets, and improving patient outcomes on a global scale.

## 6 Funding

This work is financially supported by the German 10.13039/501100002347Federal Ministry of Education and Research (BMBF) under grant number 031L0288C (Deep Legion).

## CRediT authorship contribution statement

**Oluwafemi A. Sarumi:** Writing – original draft, Resources, Conceptualization. **Dominik Heider:** Writing – review & editing, Funding acquisition.

## Declaration of Competing Interest

The authors declare that they have no known competing financial interests or personal relationships that could have appeared to influence the work reported in this paper.

## References

[1] Radford A., Narasimhan K., Salimans T., Sutskever I. (2018).

[2] Liu Y., Ott M., Goyal N., Du J., Joshi M., Chen D. (2019). A robustly optimized bert pretraining approach. https://arxiv.org/abs/1907.11692.

[3] Touvron H., Lavril T., Izacard G., Martinet X., Lachaux M.-A., Lacroix T. (2023). Llama: open and efficient foundation language models. https://arxiv.org/abs/2302.13971.

[4] Radford A., Wu J., Child R., Luan D., Amodei D., Sutskever I. (2019). Language models are unsupervised multitask learners. OpenAI Blog.

[5] Devlin J., Chang M.-W., Lee K., Toutanova K. (2019). Bert: pre-training of deep bidirectional transformers for language understanding. https://arxiv.org/abs/1810.04805.

[6] Vaswani A., Shazeer N., Parmar N., Uszkoreit J., Jones L., Gomez A.N. (2017). Attention is all you need. Adv Neural Inf Process Syst.

[7] Wolf T., Debut L., Sanh V., Chaumond J., Delangue C., Moi A., Liu Q., Schlangen D. (2020). Proceedings of the 2020 conference on empirical methods in natural language processing: system demonstrations.

[8] Brown T.B., Mann B., Ryder N., Subbiah M., Kaplan J., Dhariwal P. (2020). Proceedings of the 34th international conference on neural information processing systems, NIPS '20.

[9] Howard J., Ruder S. (2018). Proceedings of the 56th Annual Meeting of the Association for Computational Linguistics (Volume 1: Long Papers).

[10] Oniani D., Hilsman J., Zang C. (2024). Emerging opportunities of using large language models for translation between drug molecules and indications. Sci Rep.

[11] Babjac A.N., Lu Z., Emrich S.J. (2023). Proceedings of the 14th ACM international conference on bioinformatics, computational biology, and health informatics, BCB ’23.

[12] Cao Y., Liu Y., Sun H. (2023). Biobert and genebert: enhancing biological pathway analysis with pre-trained transformer models. Nat Comput Biol.

[13] Elnaggar A., Heinzinger M., Dallago C., Rehawi G., Wang Y., Jones L. (2021). Prottrans: towards cracking the language of life's code through self-supervised deep learning and high performance computing. https://arxiv.org/abs/2007.06225.

[14] Rao R., Liu J., Verkuil R., Meier J., Canny J.F., Abbeel P. (2021). 10.1101/2021.02.12.430858.

[15] Ji Y., Zhou Z., Liu H., Davuluri R.V. (2021). Dnabert: pre-trained bidirectional encoder representations from transformers model for dna-language in genome. Bioinformatics.

[16] Akiyama M., Sakakibara Y. (2022). Informative RNA base embedding for RNA structural alignment and clustering by deep representation learning. NAR Genomics Bioinform.

[17] Kulikov I., Jha D., Zhou J. (2023). Biolm: large-scale language models for biomedical text mining and genomic data integration. Bioinformatics.

[18] Sidorov P., Naulaerts S., Ariey-Bonnet J., Pasquier E., Chepelev L. (2022). Drugtarget-gpt: large language model for drug target identification. Brief Bioinform.

[19] Chen L., Lu Y., Zhao H., Wang Q. (2021). Transformercpi: improving compound-protein interaction prediction by sequence-based deep learning with self-attention mechanism and label smoothing. Brief Bioinform.

[20] Elsborg J., Salvatore M. (2023). Using llms and explainable ml to analyze biomarkers at single-cell level for improved understanding of diseases. Biomolecules.

[21] Wang L., Wang H., He H. (2023). Transforming precision medicine with large language models: enhancing individualized treatments through genomic data analysis. J Biomed Inform.

[22] Ji Y., Zhou Z., Liu H., Davuluri R.V. (2021). DNABERT: pre-trained bidirectional encoder representations from transformers model for DNA-language in genome. Bioinformatics.

[23] Brandes N., Ofer D., Peleg Y., Rappoport N., Linial M. (2022). ProteinBERT: a universal deep-learning model of protein sequence and function. Bioinformatics.

[24] Zhang D., Zhang W., Zhao Y., Zhang J., He B., Qin C. (2023). Dnagpt: a generalized pre-trained tool for versatile dna sequence analysis tasks. https://arxiv.org/abs/2307.05628.

[25] Ferruz N., Schmidt S., Höcker B. (2022). Protgpt2: a deep unsupervised language model for protein design. Nat Commun.

[26] Zhou Z., Ji Y., Li W., Dutta P., Davuluri R., Liu H. (2024). Dnabert-2: efficient foundation model and benchmark for multi-species genome. https://arxiv.org/abs/2306.15006.

[27] Zhou Z., Wu W., Ho H., Wang J., Shi L., Davuluri R.V. (2024). Dnabert-s: learning species-aware dna embedding with genome foundation models. https://arxiv.org/abs/2402.08777.

[28] Nijkamp E., Ruffolo J., Weinstein E.N., Naik N., Madani A., Xie J. (2022). Progen2: exploring the boundaries of protein language models. https://arxiv.org/abs/2206.13517.

[29] Jumper J., Evans R., Pritzel A., Green T., Figurnov M., Ronneberger O. (2021). Highly accurate protein structure prediction with alphafold. Nature.

[30] Chen M., Tworek J., Jun H., Yuan Q., Pinto H.P.d.O., Kaplan J. (2021). Evaluating large language models trained on code. https://arxiv.org/abs/2107.03374.

[31] Park M., Seo S., Park E., Kim J. (2022). Epibertope: a sequence-based pre-trained bert model improves linear and structural epitope prediction by learning long-distance protein interactions effectively. bioRxiv.

[32] Rives A., Meier J., Sercu T., Goyal S., Lin Z., Liu J. (2019). Biological structure and function emerge from scaling unsupervised learning to 250 million protein sequences. Proc Natl Acad Sci.

[33] Meier J., Rao R., Verkuil R., Liu J., Sercu T., Rives A. (2021). Language models enable zero-shot prediction of the effects of mutations on protein function. bioRxiv.

[34] Hsu C., Verkuil R., Liu J., Lin Z., Hie B., Sercu T. (2022). Learning inverse folding from millions of predicted structures. 10.1101/2022.04.10.487779.

[35] Lin Z., Akin H., Rao R. (2022). Evolutionary-scale prediction of atomic level protein structure with a language model. 10.1101/2022.07.20.500902.

[36] Mo S., Fu X., Hong C., Chen Y., Zheng Y., Tang X. (2021). Multi-modal self-supervised pre-training for regulatory genome across cell types. https://arxiv.org/abs/2110.05231.

[37] Wu H., Zhang Y., Wang W. (2021). International conference on learning representations (ICLR).

[38] Refahi M., Sokhansanj B., Rosen G. (2023). 2023 IEEE signal processing in medicine and biology symposium (SPMB).

[39] Nguyen E., Poli M., Faizi M., Thomas A., Birch-Sykes C., Wornow M. (2023). Hyenadna: long-range genomic sequence modeling at single nucleotide resolution. https://arxiv.org/abs/2306.15794.

[40] Wu Z., Jiang D., Wang J., Zhang X., Du H., Pan L. (2022). Knowledge-based bert: a method to extract molecular features like computational chemists. Brief Bioinform.

[41] Wang S., Guo Y., Wang Y., Sun H. (2019). Smiles-bert: large scale unsupervised pre-training for molecular property prediction. https://arxiv.org/abs/1907.10903.

[42] Chithrananda S.A., Grand G., Ramsundar B. (2020). Chemberta: large-scale self-supervised pretraining for molecular property prediction. https://arxiv.org/abs/2010.09885.

[43] Valizadeh Aslani T., Shi Y., Ren P., Wang J., Zhang Y., Hu M. (2023). PharmBERT: a domain-specific BERT model for drug labels. Brief Bioinform.

[44] Kalakoti Y., Chawla K., Li X., Zhang Q., Zhang L., Wang L. (2022). Transdti: transformer-based language models for estimating drug-target interactions and building a drug recommendation workflow. Bioinformatics.

[45] Bagal V., Aggarwal R., Gupta S., Arora A. (2022). Molgpt: molecular generation using a transformer-decoder model. J Cheminform.

[46] Joachimiak M.P., Caufield J.H., Harris N.L., Kim H., Mungall C.J. (2023). Gene set summarization using large language models. https://arxiv.org/abs/2305.13338.

[47] Wu Z. (2022). Bingo: a large language model- and graph neural network-based workflow for the prediction of essential genes from protein data. Brief Bioinform.

[48] Truhn D., Eckardt J.N., Ferber D. (2024). Large language models and multimodal foundation models for precision oncology. npj Prec Oncol.

[49] Sorin V., Barash Y., Konen E. (2023). Large language models for oncological applications. J Cancer Res Clin Oncol.

[50] Khan A., Lee B. (2024). Deepgene transformer: transformer for the gene expression-based classification of cancer subtypes. https://arxiv.org/abs/2108.11833.

[51] Toufiq M., Rinchai D., Bettacchioli E., Kabeer B., Khan T., Subba B. (2023). Harnessing large language models (llms) for candidate gene prioritization and selection. J Transl Med.

[52] Yang F., Wang W., Wang F. (2022). Scbert as a large-scale pretrained deep language model for cell type annotation of single-cell rna-seq data. Nat Mach Intell.

[53] Ruan A., Mantravadi A., George C. (2021). GeneBERT: BERT for predicting differential gene expression from histone modifications. https://github.com/ZovcIfzm/GeneBERT.

[54] Luo H., Chen C., Shan W., Ding P., Luo L. (2022). Intelligent Computing Theories and Application: 18th International Conference, ICIC 2022, Xi'an, China, August 7–11, 2022, Proceedings, Part II.

[55] Zeng W., Gautam A., Huson D.H. (2022). Mulan-methyl—multiple transformer-based language models for accurate dna methylation prediction. GigaScience.

[56] Azam M., Chen Y., Arowolo M.O., Liu H., Popescu M., Xu D. (2024). A comprehensive evaluation of large language models in mining gene interactions and pathway knowledge. 10.1101/2024.01.21.576542.

[57] Wong C.-K., Choo A., Cheng E.C.C., San W.-C., Cheng K.C.-K., Lau Y.-M. (2024). Lomics: generation of pathways and gene sets using large language models for transcriptomic analysis. https://arxiv.org/abs/2407.09089.

[58] Park G., Yoon B.-J., Luo X., Lopez-Marrero V., Demner-Fushman Dina, Ananiadou Sophia, Cohen Kevin (jul. 2023). The 22nd Workshop on Biomedical Natural Language Processing and BioNLP Shared Tasks.

[59] Lee J., Yoon W., Kim S., Kim D., Kim S., So C.H. (2019). Biobert: a pre-trained biomedical language representation model for biomedical text mining. Bioinformatics.

[60] Alsentzer E., Murphy J., Boag W., Weng W.-H., Jindi D., Naumann T., Rumshisky A., Roberts K., Bethard S., Naumann T. (2019). Proceedings of the 2nd clinical natural language processing workshop, association for computational linguistics.

[61] Wu C., Schwartz J.-M., Nenadic G. (2013). Pathner: a tool for systematic identification of biological pathway mentions in the literature. BMC Syst Biol.

[62] Zhao H., Chen H., Yang F., Liu N., Deng H., Cai H. (2023). Explainability for large language models: a survey. https://arxiv.org/abs/2309.01029.

[63] Mehrabi N., Morstatter F., Saxena N., Lerman K., Galstyan A. (2021). A survey on bias and fairness in machine learning. ACM Comput Surv.

[64] Wang C., Liu S., Yang H., Guo J., Wu Y., Liu J. (2023). Ethical considerations of using chatgpt in health care. J Med Internet Res.

[65] Hatem R., Simmons B., Thornton J.E. (2023). A call to address ai “hallucinations” and how healthcare professionals can mitigate their risks. Cureus.

[66] Goddard J. (2023). Hallucinations in chatgpt: a cautionary tale for biomedical researchers. Am J Med.

